# Supplementary material for: Paracoccidioidomycosis in people living with HIV/AIDS: A historical retrospective cohort study in a national reference center for infectious diseases, Rio de Janeiro, Brazil
Source: PLoS Negl Trop Dis. 2022 Jun 15;16(6):e0010529. doi: 10.1371/journal.pntd.0010529 (PMC9239448; doi:10.1371/journal.pntd.0010529)
Supplement: Attachment — Submitted filename: rebuttalletter.docx [file pntd.0010529.s002.docx]

Evandro Chagas National Institute of Infectious Diseases, INI/Fiocruz

Avenida Brasil, 4365, Manguinhos

CEP 21040-900 - Rio de Janeiro - RJ - BRAZIL

Phone: +55 (21) 38659578

E-mail: eduardo.falcao@ini.fiocruz.br

May 4^th^, 2022

Dear Academic Editor and Reviewer,

We sincerely appreciate the thoughtful review provided for our manuscript “Paracoccidioidomycosis in people living with HIV/AIDS: a historical retrospective cohort study in a national reference center for infectious diseases, Rio de Janeiro, Brazil” (PNTD-D-22-00221). Addressing the constructive comments has improved our article. All suggestions were considered and we managed to answer and solve all the possible issues. Our point-by-point responses to the Reviewers’ comments can be found below. Please consider the file with tracked changes to see the lines indicated in the answers.

Reviewer #1:

1-The abstract needs to be improved and must include more data related with the study.

**Thank you for the suggestion. We made some changes and included more data.**

2-The Key words were not described.

**Indeed. We added the keywords. Please see line 24.**

3-The Results: They are very extensive, repetitive in both text and tables.

**We agree. We made some changes in the Results sections to reduce duplicate information being more concise.**

4-The Tables could be condensed in one or two.

**Thank you for this contribution. We grouped Tables 2 and 3 together and made the corresponding changes in the figure legends and within the text. Please see lines 197 and 219-232.**

5- The presented figures, maybe the number two has more relevance to be included into the paper. the others could be removed.

**We understand the Reviewer’s point. However, for the sake of the readers, the authors consider that all figures included are useful to represent the wide (and sometimes atypical) clinical spectrum of PCM in PLWHA. So, the authors would rather keep all figures.**

6-The discussion section is very extensive, few adequated and needs to be more concise and focalized on HIV/ PCM coinfection together with the literature data.

**We agree and appreciate the Reviewer’s comment. We've made several changes in the Discussion section focusing on the HIV/PCM coinfection and we think these alterations made the content more clear, fluid and concise. Please see lines 285-288, 307-311, 325-332, 361-371, 389-394.**

7-Generalizations are used with frequency and they must be avoided, due to the small size of the individuals included.
**We agree and this is an important point. Despite the large cohort samples of PCM and HIV in our center and the long period studied, PCM/HIV confection is indeed uncommon, as observed in other case series. Therefore, the number of patients included in these studies is a limiting factor to allow general conclusions. The authors made some changes within the text to avoid generalizations. Please see lines 275, 291, 304, 315.**

Minor comments.

1-Please verify reference number 12. It is inadequate for citation. See Almeida et al 2017.Benard et al 2000. Some of the references are few originals

**Thank you for this correction. We changed it to Almeida et al 2017. In addition we included a new reference (de Almeida JR et al 2019).**

2-The paragraph between lines 87-90 is inadeaquate.

**The authors agree and corrected it. Please see lines 93-98.**

3-There is a contradiction in values related with the prevalence of the acute clinical form of PCM <10% line 281, and 26% line288.

**Indeed. We corrected it. Please see lines 307-317.**

4-It would be interesting to cite the reference which describe 12% of PCM in HIV infected patients Line 90.

**Indeed. The reference is Sarti et al. 2012, we corrected it. Please see lines 93-98.**

5-It is very importan to clarify why the high variability of therapy time in these patients From 12 to 60 months?This is very different from elsewere

**This is another important contribution of the Reviewer. Thank you for this observation. We have included a stretch to clarify this important point in this new version of the manuscript. Please see lines 389-394.**

6-I suggest to improve the information and disccussion of the two cases who developed IRIS. This is an exciting matter to give an upgrade in your manuscript.

**Thank you. The authors agree and consider that this comment contributed to improve the quality of our manuscript. Please see lines 181-191 and 318-332.**

7-The English style must be reviewed.

**While revising the manuscript, we made several changes and also revised the English style again. The authors consider that this new version fulfills a standard academic language, comprehensive to the readers and is suitable for publication in PNTD.**

Yours sincerely,

**Eduardo Mastrangelo Marinho Falcão - MD, MSc**
